# Effect modification of environmental factors on influenza-associated mortality: a time-series study in two Chinese cities

**DOI:** 10.1186/1471-2334-11-342

**Published:** 2011-12-14

**Authors:** Lin Yang, Ping Yan Chen, Jian Feng He, King Pan Chan, Chun Quan Ou, Ai Ping Deng, JS Malik Peiris, Chit Ming Wong

**Affiliations:** 1 Department of Community Medicine, School of Public Health, The University of Hong Kong, Hong Kong Special Administrative Region, 5/F William Mong Block, 21 Sassoon Road, Hong Kong, China; 2 Department of Biostatistics, School of Public Health and Tropical Medicine, Southern Medical University, Guangzhou, China; 3 Guangdong Provincial Centre for Disease Control and Prevention, Guangzhou, China; 4 School of Public Health, The University of Hong Kong, Pokfulam, Hong Kong Special Administrative Region, China; 5 HKU Pasteur Research Centre, Pokfulam, Hong Kong Special Administrative Region, China

## Abstract

**Background:**

Environmental factors have been associated with transmission and survival of influenza viruses but no studies have ever explored the role of environmental factors on severity of influenza infection.

**Methods:**

We applied a Poisson regression model to the mortality data of two Chinese metropolitan cities located within the subtropical zone, to calculate the influenza associated excess mortality risks during the periods with different levels of temperature and humidity.

**Results:**

The results showed that high absolute humidity (measured by vapor pressure) was significantly (*p *< 0.05) associated with increased risks of all-cause and cardiorespiratory deaths, but not with increased risks of pneumonia and influenza deaths. The association between absolute humidity and mortality risks was found consistent among the two cities. An increasing pattern of influenza associated mortality risks was also found across the strata of low to high relative humidity, but the results were less consistent for temperature.

**Conclusions:**

These findings highlight the need for people with chronic cardiovascular and respiratory diseases to take extra caution against influenza during hot and humid days in the subtropics and tropics.

## Background

Influenza used to be considered as a "cold" disease as it usually returns every cold winter in temperate countries. However, recent studies have shown that influenza can be active throughout the year in the warm tropics and subtropics and the disease burden of influenza there can be as heavy as that in temperate climates [1-4]. It has been proposed that influenza seasonality is driven by complicated interactions between antigenic drifts of virus strains, environmental factors, host susceptibility and behavior changes [5-7]. Among these potential factors, environmental factors including temperature and relative humidity have been most thoroughly explored by laboratory and observational studies [8,9]. Recent studies raised a hypothesis that absolute humidity is one of drivers for influenza seasonality in temperate regions [10,11], but the mechanism behind various seasonal patterns of influenza outbreaks under different climates remains unclear.

**Figure 1:**
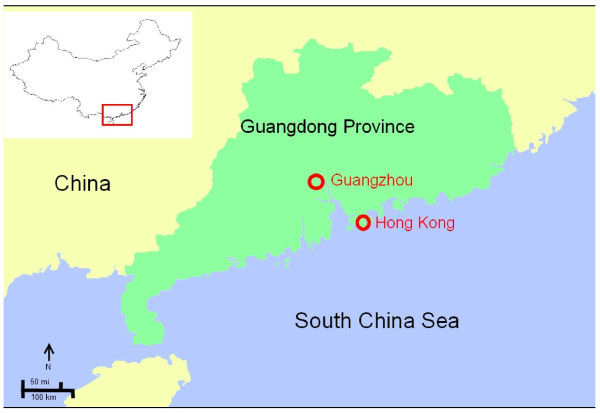
**Map of Guangzhou and Hong Kong**.

Given the association of environmental factors with both influenza virus activity and mortality, these factors have been adjusted for as confounders in the statistical models for influenza associated mortality burden [2,12]. However, few of previous laboratory or epidemiological studies tackled the potential modifying role of environmental factors on severity of infection, or mortality risks associated with influenza. Such effect modification is plausible because extreme weather conditions could have a synergistic effect with influenza on mortality burden. Both cold and hot temperatures have been associated with increased mortality risks of respiratory diseases in numerous studies [13-15]. Our previous study in Hong Kong also found a two-peak seasonal variation in mortality burden of influenza, which is similar to the pattern of influenza seasonality, suggesting that environmental factors might also affect the severity of seasonal influenza infection [16]. In this study we applied a Poisson regression model to the data of two subtropical Chinese cities: Guangzhou and Hong Kong, to examine the possible effect modification of environmental factors on the severity of influenza infection measured by the mortality attributable to influenza. These two cities are geographically close, with Guangzhou located at latitude 23°N and Hong Kong at 21°N (Figure 1). Both cities have a typical subtropical climate, but on average Hong Kong has a higher temperature and humidity. Hong Kong also has a larger population than Guangzhou (6.8 million vs. 3.7 million).We examined three environmental factors which have been documented to regulate virus survival and transmission: temperature, relative humidity and absolute humidity.

## Methods

### Data

Influenza surveillance data were obtained from the Hong Kong Department of Health (DH) for the period of 1998-2006 in Hong Kong and the Guang Dong Provincial Centre for Disease Control and Prevention (GDCDC) for 2004-2006 in Guangzhou [17]. In both cities the consultation rates for influenza-like illness (ILI) from sentinel hospitals or doctors were reported on the weekly basis, with diagnosis criteria of fever (≥38°C), cough or sore throat. Nasopharyngeal aspirate or swab was collected from ILI patients and then tested of influenza viruses by immunofluorescence and cell culture. Positive specimens were further typed (subtyped) into A/H3N2, A/H1N1 and B by haemagglutination inhibition test with strain-specific antisera provided by the World Health Organization. During 2004-2006, annual averages of 35,256 and 1,872 specimens were tested by the DH and GDCDC, respectively.

Mortality data for the period of 2004-2006 were obtained from the Hong Kong Census and Statistics Department and the Guangzhou Department of Health. According to Hong Kong Law all deaths from natural causes are required to be registered, so the mortality data in Hong Kong was fairly complete. The registered death data collected by the Guangzhou Department of Health covered the residents from the eight urban districts, but did not include the immigrant population and residents living in the two suburban districts. Therefore, the population denominator of Guangzhou did not change much during the study period. Given the fact that most immigrant workers are young adults and few died in Guangzhou, we can assume that our mortality data was complete and representative of the general population of Guangzhou. We aggregated the weekly numbers of deaths with underlying cause of cardiorespiratory disease, pneumonia and influenza, and all-cause mortality. The corresponding International Classification of Diseases, Tenth Revision (ICD10) codes adopted by Guangzhou and Hong Kong are I00-I99, J00-J99 for cardiorespiratory, J10-J18 for pneumonia and influenza, and A00-R99 for all-cause deaths. We used the accidental deaths (ICD10 codes S00-T989) in Hong Kong as the control disease. Weekly mean temperature and relative humidity were separately derived from the National Meteorological Information Centre of China and Hong Kong Observatory for Guangzhou and Hong Kong respectively. Weekly mean vapor pressure was calculated as a metric for absolute humidity following an equation provided by Basu et al. [18],

(1) $$\text{vapor}\;\text{pressure}=0.0\text{6112}*\text{relative}\;\text{humidity}\%*\text{1}0^{\text{7}.\text{5}*\text{temperature}\left({}^{\circ}\text{C}\right)/(\text{237}.\text{7}+\text{temperature}({}^{\circ}\text{C}))}$$

Vapor pressure will be used for absolute humidity hereafter in this paper.

### Statistical methods

A generalized additive model (GAM) with a log link function and Poisson error was fitted to the weekly numbers of deaths. The Poisson model has been widely applied to the estimation of influenza associated disease burden and recently been validated by an empirical dataset of laboratory confirmed influenza cases [19]. First, long-term trends and seasonal patterns of cause-specific mortality counts, as well as environmental factors including temperature and relative humidity, were adjusted for as confounders by building a core model:

(2) $$Log\left(y_{t}\right)=ns\left(temp_{t}\right)+ns\left(humd_{t}\right)+ns\left(t\right)$$

Here *y*_*t *_denotes the number of deaths at week *t*. *ns(t), ns(temp*_*t*_*), *and *ns(humd*_*t*_*) *denote the natural cubic spline smoothing functions of time, weekly average temperature and relative humidity. The natural spline smoothing function was used to remove the small variation while maintaining the major trend of each variable, i.e. to make them smoother. The aim of smoothing was to increase the efficiency in estimating the model coefficients [20]. Given the high correlation between temperature and vapor pressure, the smoothing function of vapor pressure was not added in order to avoid collinearity between the variables. The adequacy of this core model was evaluated by the absence of any obvious pattern in partial autocorrelation functions of its residuals (Additional file 1: Figure S1). Second, the weekly proportions of specimens positive for influenza A or B were then added into the core model as a variable for influenza virus activity to obtain a main effect model.

(3) $$Log\left(y_{t}\right)=\beta *flu_{t}+ns\left(temp_{t}\right)+ns\left(humd_{t}\right)+ns\left(t\right)$$

The main effects of influenza have been presented elsewhere [21]. To explore the effect modification of environmental factors on influenza associated mortality, we added into the core model the product terms of influenza proportion variable and dummy variables for periods of normal and extreme (high or low) weather conditions as interaction terms between virus activity and environmental factors. For example, the interaction model for temperature and influenza mortality was:

(4) $$\begin{array}{c} Log\left(y_{t}\right)=\beta_{\text{1}}flu_{t}*Lowtemp_{t}+\beta_{\text{2}}flu_{t}*Midtemp_{t}+\beta_{\text{3}}flu_{t}*Hightemp_{t}\\ +ns\left(temp_{t}\right)+ns\left(humd_{t}\right)+ns\left(t\right) \end{array}$$

where *Lowtemp*_*i *_= 1 for the periods within low temperature ranges and 0 for otherwise, and *Midtemp*_*t *_and *Hightemp*_*t *_are similarly defined as the dummy variables for the middle and high temperature periods. The smoothing function of temperature *ns(temp*_*t*_*) *was kept in the model in order to adjust for the association of temperature and mortality. It is reasonable to assume that the cutoff point of extreme weather may differ across cities as people may adapt to prevailing climates. We used the first (25%) and third quartiles (75%) of weekly average temperatures (or humidity) as the cutoff points to define the low, middle and high temperature (or humidity) periods [22]. The presence of effect modification by environmental factors was evaluated using likelihood ratio tests between the interaction and main effect models. To measure the effects of influenza on mortality, we computed the percentage change of mortality counts associated with 1% increases of influenza virus activity for the low, middle and high periods. The formula for the low temperature period is

(5) $$\%change=\left[\text{exp}\left(0.0\text{1}*\beta_{\text{1}}\right)-\text{1}\right]*\text{1}00$$

Here β_1 _was obtained from the above interaction model. To test whether our results were robust to various definitions of periods, we chose two sets of extra cutoff points: 20th and 80th, 30th and 70th percentiles of weekly average data in each city. Although we removed the autocorrelation and seasonal trends within mortality data, there are still concerns that such adjustment was inadequate and the remaining uncontrolled seasonal factors may cause interaction terms of environmental factors and virus activity to appear significant in our models. To rule out this possibility, we used accidental deaths that were expected to be unrelated to influenza infection as a control mortality group to show that our findings are unlikely the spurious results of under-adjustment of seasonal confounding factors in modeling. Some previous studies used the anomalies to assess the effects of meteorology factors on influenza [11].We therefore conducted a sensitivity analysis by defining the strata by anomalies, instead of absolute values of meteorology factors. The anomalies were defined as the deviations of observed metrological data from a seasonal curve with a constant and a sinusoidal pair fitted to these observed data. All the analyses were performed using the mgcv package of R software (version 2.5.1.) [23].

## Results

**Figure 2:**
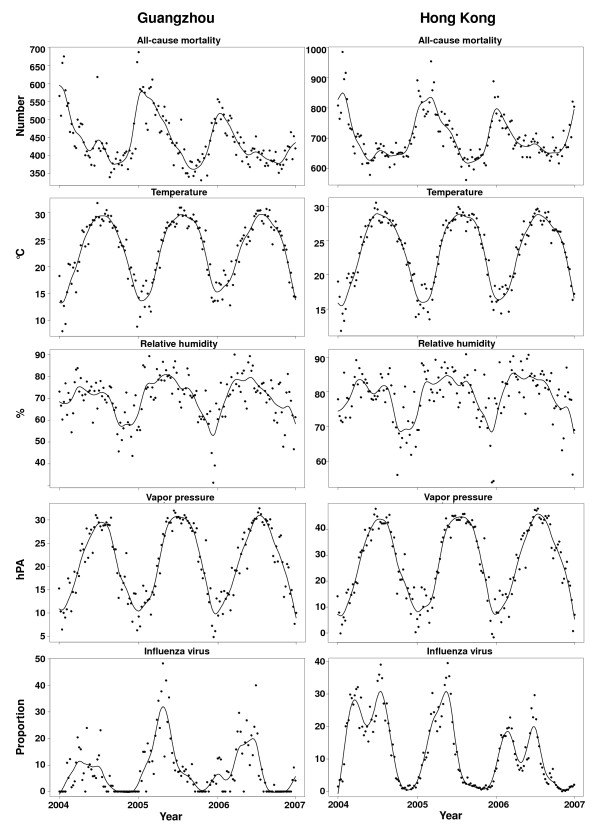
**Time series plots of all-cause mortality, temperature, relative humidity and proportions of influenza positive specimens**. Observed data (*dots*) are smoothed by a natural cubic spline with 8 degrees of freedom per year (*line*).

Hong Kong has a larger population than Guangzhou (6.8 million versus 3.7 million) during our study period. On average, Hong Kong has a slightly higher temperature, relative humidity and vapor pressure, and smaller annual variations than Guangzhou (Table 1). The mean of the weekly proportions of specimens positive for influenza A or B was higher in Hong Kong than in Guangzhou. In 2004 and 2006, there were two peaks of virus activity in Hong Kong (one in February/March and another in June/July), but only one broad peak in Guangzhou (Figure 2). The influenza seasonality was similar between these two cities in 2005.

**Table 1:** Descriptive statistics for weekly average temperature, relative humidity, vapor pressure and counts of all-cause deaths

				Percentile				
**Variable**	**City**	**Mean**	**SD**	**min**.	**25**	**50**	**75**	**max**

Temperature (°C)	Guangzhou	23.0	5.8	8.1	18.4	24.5	27.8	31.7
	
	Hong Kong	23.4	4.9	11.8	19.2	24.8	27.7	30.5

Relative humidity (%)	Guangzhou	70.0	10.3	31.4	63.6	71.8	76.8	89.9
	
	Hong Kong	78.9	7.5	53.9	75.5	80.0	84.6	90.9

VP (hPA)	Guangzhou	20.9	7.7	4.9	14.2	21.0	28.5	32.5
	
	Hong Kong	23.7	7.1	9.2	17.5	24.5	30.7	34.0

All-cause deaths (< 65 years)	Guangzhou	110	15	85	99	110	118	179
	
	Hong Kong	147	13	116	138	147	156	186

All-cause deaths (≥65 years)	Guangzhou	333	64	237	287	316	371	556
	
	Hong Kong	555	72	431	502	538	596	813

More deaths with underlying causes of cardiorespiratory, pneumonia and influenza or all-cause were recorded in the low temperature (or low vapor pressure) periods for both Guangzhou and Hong Kong (Table 2). For both cities, the mortality counts did not show any obvious difference across levels of relative humidity. High influenza virus activity coincided with high levels of temperature, relative humidity or vapor pressure, with the only exception of Guangzhou which had higher mean proportions when temperature is within the middle range.

**Table 2:** Weekly mean of mortality numbers and proportions of specimens positive for influenza (virus %) during the periods with low, middle and high levels of environmental factors

		Mean (SD)		
	**City**	**Low**	**Middle**	**High**

			**Temperature**	

All-cause	Guangzhou	520.5 (81.4)	424.0 (52.1)	407.3 (49.0)
	
	Hong Kong	798.6 (73.5)	677.5 (48.6)	655.9 (36.6)

CRD	Guangzhou	300.5 (61.6)	227.1 (43.6)	213.4 (37.4)
	
	Hong Kong	382.7 (47.5)	296.4 (35.3)	275.6 (25.4)

P&I	Guangzhou	11.3 (4.9)	9.7 (4.3)	9.5 (3.3)
	
	Hong Kong	90.8 (15.1)	73.6 (14.1)	72.1 (11.9)

Accident	Hong Kong	28.0 (7.6)	27.8 (6.9)	28.2 (5.2)
	
Virus %	Guangzhou	5.6 (5.6)	9.2 (11.9)	7.7 (8.4)

	Hong Kong	11.1 (9.1)	12.1 (11.7)	13.8 (10.8)
	
			**Relative humidity**	

All-cause	Guangzhou	441.9 (87.7)	441.0 (72.9)	451.9 (64.2)
	
	Hong Kong	722.6 (92.4)	693.7 (71.4)	699.7 (71.1)

CRD	Guangzhou	241.1 (65.5)	239.9 (56.7)	247.1 (54.8)
	
	Hong Kong	328.0 (65.6)	306.6 (51.2)	309.9 (49.5)

P&I	Guangzhou	9.3 (4.1)	10.1 (4.1)	10.6 (4.6)
	
	Hong Kong	76.9 (16.8)	77.5 (16.0)	78.0 (14.7)

Accident	Hong Kong	26.5 (7.7)	28.3 (5.8)	28.7 (7.1)
	
Virus %	Guangzhou	2.0 (3.3)	7.1 (8.2)	15.8 (12.2)

	Hong Kong	4.3 (6.4)	14.8 (10.9)	15.2 (10.6)
	
			**Vapor pressure**	

All-cause	Guangzhou	498.2 (91.7)	432.1 (59.8)	413.4 (53.0)
	
	Hong Kong	790.8 (79.4)	680.6 (53.8)	657.7 (36.1)

CRD	Guangzhou	284.4 (68.8)	233.2 (48.2)	217.2 (42.1)
	
	Hong Kong	376.0 (53.7)	299.1 (39.0)	277.0 (25.2)

P&I	Guangzhou	10.6 (5.1)	9.9 (3.6)	9.6 (4.5)
	
	Hong Kong	89.2 (16.5)	74.3 (14.3)	72.1 (11.8)

Accident	Hong Kong	28.2 (7.8)	28.0 (6.7)	27.6 (5.4)
	
Virus %	Guangzhou	4.3 (5.3)	8.3 (10.5)	10.8 (11.1)

	Hong Kong	9.9 (9.4)	12.1 (11.2)	14.9 (11.3)

*SD *standard deviation; *CRD *cardiorespiratory; *P&I *pneumonia and influenza

Significant interaction between temperature and influenza on the mortality risks was only found in cardiorespiratory mortality in Hong Kong (*p *< 0.05), and the interaction between relative humidity and influenza was found significant for all-cause mortality in both Guangzhou and Hong Kong, and for cardiorespiratory mortality only in Guangzhou (Table 3). The patterns of influenza impact across the low to high temperature periods were not consistent among the two cities. In Guangzhou, the highest risk of mortality tended to be observed in the middle-temperature period, whereas for Hong Kong the largest changes in risk were found in the periods with high temperature for the three mortality categories (Table 3). An increasing pattern of influenza associated mortality risks could be observed along low-, mid- and high-relative humidity periods in Guangzhou and Hong Kong, but most of estimates for low- and high-periods were not statistically significant (Table 3).

**Table 3:** Percentage change (%) of mortality counts associated with 1% increase in influenza virus activity during the low, middle and high periods of temperature, relative humidity and vapor pressure

Disease	City	Percentage change (95% CI)			Deviance	Deviance	*p*-value*
		**Low**	**Middle**	**High**	**(main)**	**(interaction)**	

		**Temperature**					

All-cause	Guangzhou	0.02 (-0.50, 0.54)	0.29 (0.07, 0.50)	0.26 (-0.11, 0.63)	608.3	603.5	0.089
	
	Hong Kong	0.11 (-0.08, 0.30)	0.15 (0.01, 0.28)	0.24 (0.06, 0.41)	343.9	340.6	0.187

CRD	Guangzhou	0.15 (-0.56, 0.86)	0.41 (0.11, 0.72)	0.50 (-0.03, 1.03)	642.2	638.9	0.188
	
	Hong Kong	0.13 (-0.14, 0.39)	0.27 (0.08, 0.47)	0.49 (0.24, 0.74)	320.8	310.7	0.006

P&I	Guangzhou	0.14 (-1.71, 2.03)	1.37 (0.64, 2.09)	0.82 (-0.41, 2.06)	189.3	186.1	0.201
	
	Hong Kong	0.38 (-0.11, 0.86)	0.66 (0.33, 1.00)	0.79 (0.36, 1.23)	241.2	238.0	0.203

Accident	Hong Kong	0.32 (-0.34, 0.98)	0.20 (-0.24, 0.63)	0.11 (-0.44, 0.67)	159.1	158.9	0.871
	
		**Relative humidity**					

All-cause	Guangzhou	-0.60 (-1.50, 0.32)	0.13 (-0.13, 0.40)	0.44 (0.20, 0.68)	608.3	579.8	< 0.001
	
	Hong Kong	0.04 (-0.26, 0.33)	0.13 (0.01, 0.25)	0.29 (0.12, 0.46)	343.9	333.7	0.006

CRD	Guangzhou	-0.79 (-2.04, 0.48)	0.32 (-0.05, 0.70)	0.57 (0.23, 0.91)	642.2	621.3	< 0.001
	
	Hong Kong	0.22 (-0.18, 0.62)	0.27 (0.09, 0.44)	0.41 (0.16, 0.66)	320.8	317.4	0.178

P&I	Guangzhou	-1.67 (-4.95, 1.74)	0.89 (-0.02, 1.81)	1.66 (0.85, 2.48)	189.3	183.5	0.055
	
	Hong Kong	0.52 (-0.22, 1.26)	0.60 (0.29, 0.91)	0.76 (0.34, 1.18)	241.2	240.1	0.570

Accident	Hong Kong	0.91 (0.00, 1.83)	0.13 (-0.27, 0.53)	0.26 (-0.29, 0.81)	159.1	155.3	0.144
	
		**Vapor pressure**					

All-cause	Guangzhou	-0.20 (-0.75, 0.35)	0.27 (0.04, 0.51)	0.35 (0.07, 0.62)	608.3	594.0	0.001
	
	Hong Kong	0.13 (-0.07, 0.32)	0.11 (-0.03, 0.25)	0.26 (0.09, 0.42)	343.9	336.7	0.027

CRD	Guangzhou	-0.06 (-0.81, 0.70)	0.38 (0.05, 0.71)	0.54 (0.16, 0.93)	642.2	632.1	0.006
	
	Hong Kong	0.14 (-0.13, 0.41)	0.24 (0.04, 0.44)	0.49 (0.25, 0.72)	320.8	309.6	0.004

P&I	Guangzhou	-0.05 (-2.03, 1.98)	1.04 (0.23, 1.86)	1.58 (0.73, 2.44)	189.3	185.8	0.172
	
	Hong Kong	0.45 (-0.04, 0.93)	0.71 (0.36, 1.06)	0.67 (0.26, 1.08)	241.2	239.3	0.391

Accident	Hong Kong	0.49 (-0.16, 1.14)	0.23 (-0.22, 0.68)	-0.02 (-0.54, 0.50)	159.1	157.3	0.392

*CRD *cardiorespiratory; *P&I *pneumonia and influenza; *CI *confidence interval

* *p*-value was derived from the likelihood ratio test between the interaction and main effect model

The interaction between vapor pressure levels and virus activity were found to be significant (*p *< 0.05) for all-cause and cardiorespiratory mortality, but not for pneumonia and influenza mortality (Table 3). Consistently higher mortality risks were found at the high levels of vapor pressure, with only exception of P&I mortality in Hong Kong. The influenza effects on mortality of cardiorespiratory, pneumonia and influenza, and all causes were found significant during the middle- and high-vapor pressure periods (with the only exception of all-cause mortality in Hong Kong), but not significant when vapor pressures remained at the relatively low levels. For the high vapor pressure periods, all-cause excess mortality counts attributable to influenza would increase by 0.35% and 0.26% for per 1% increase of virus activity, and the corresponding increases for cardiorespiratory mortality were 0.54% and 0.49% for Guangzhou and Hong Kong, respectively (Table 3).

**Figure 3:**
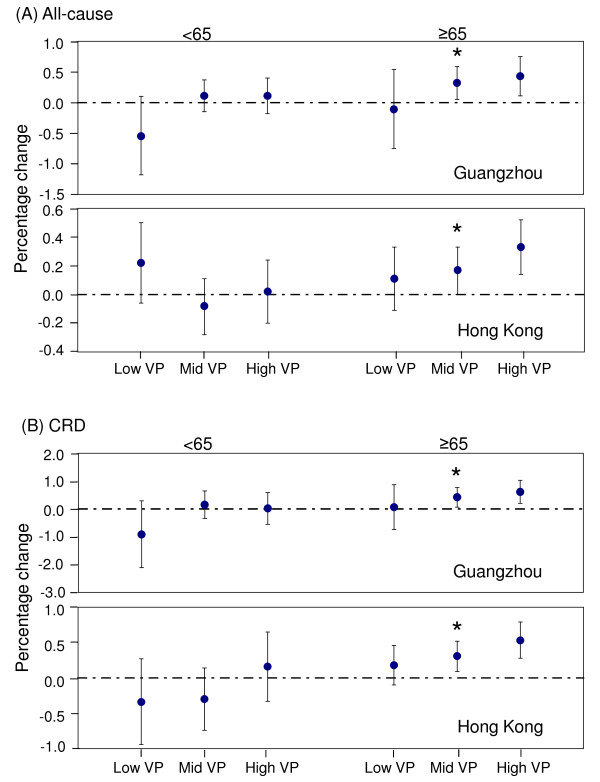
**Percentage change (%) of mortality counts for all-cause and cardiorespiratory (CRD) mortality**. The risks associated with 1% increase in influenza virus activity during the low-, middle- and high-vapor pressure periods were plotted for the age groups younger than 65 years (< 65) and equal or over 65 years (≥65). The 95% confidence intervals were shown in vertical bars. An asterisk is added above the bars if the interaction between vapor pressure and influenza is shown statistically significant by the likelihood ratio test between main effects and interaction models.

We also assessed the effect modification of environmental factors on influenza effects (i.e. interaction of each factor and influenza) for the age group younger than 65 years (< 65) and the elderly aged 65 years or older (≥65). The results of the ≥65 age group were consistent with those for the all-ages group, with an increasing trend over vapor pressure levels observed for all-cause and cardiorespiratory mortality in the two cities (Figure 3). For the < 65 age group, this trend could also be observed in most city-specific disease categories, with the only exception of all-cause mortality in Hong Kong. But the interaction terms were statistically significant (*p *< 0.05) only in the ≥65 group. The modifying effects of temperature and relative humidity on influenza effects were found quite similar between the all-ages and ≥65 age groups, but not between the all-ages and < 65 age groups (data not shown).

The models with indicators for different cutoff points returned similar estimates for temperature, relative humidity and vapor pressure (Additional file 1: Figure S2). We did not observe any significant interaction between influenza and environmental factors in terms of their effects on the control mortality category of accidental mortality in Hong Kong. Influenza associated accidental mortality risks were also not statistically significant.

To ensure the standardized comparison between Guangzhou and Hong Kong, we decided to apply the same modeling approach to the data of same study period, because the core model would have been slightly different if we used the longer time series of Hong Kong. To check the robustness of our conclusions, we repeated the above analysis using a longer time series data of Hong Kong during 1998-2006 and the estimates were shown in Additional file 1: Table S1. The statistical significance of interaction terms was close to those from the study period of 2004-2006. The estimates for the low temperature periods became larger and statistically significant; those for the high relative humidity periods were smaller and comparable to the middle relative humidity; and for the low vapor pressure periods, the estimates were similar but became significant. The increasing trend across the low, middle and high levels of temperature and relative humidity was less evident.

The results of stratification analysis by anomalies are shown in Additional file 1: Table S2. The estimates were similar to those for the periods defined by absolute values of meteorological factors, in terms of magnitude and changing patterns. But the likelihood ratio tests showed more significant interaction for temperature or vapor pressure, and less for relative humidity.

## Discussion

In this study we quantified influenza associated mortality risks at the various ranges of temperature and humidity, and compared the results between two large subtropical cities. An increasing pattern of influenza-associated mortality risks on all-cause and cardiorespiratory along the low to high periods was found for temperature, relative and absolute humidity in both cities during the period of 2004-2006. The interaction between vapor pressure indicators and virus activity were also consistently significant for relative and absolute humidity. Although lower vapor pressure (or temperature) has been found to facilitate virus transmission and survival in the guinea pig model [10], our results suggested that higher vapor pressure (or temperature) was associated with a higher mortality risk attributable to influenza. Severity of seasonal influenza epidemics were not only determined by virus transmission efficiency and outdoor weather conditions, but also largely affected by host resistance, indoor living environment and social behavior [5]. The high vapor pressure periods coincided with the summer peaks of influenza in both Guangzhou and Hong Kong, indicating that influenza could pose a higher risk when reaching its peak in seasons with high vapor pressure in subtropical cities.

Our results may help to interpret our previous findings that the effects of influenza were significantly higher in the humid and warm spring/summer period than in the dry and cold winter period in Hong Kong [16]. The extreme low vapor pressure was usually recorded during December-January and the highest appeared during June-July, which coincided with the trough and peak periods of excess risks associated with influenza viruses. Effect modification of environmental factors was only detected in all-cause and cardiorespiratory mortality, but not in pneumonia and influenza, suggesting that the synergistic interaction between high humidity (or temperature) and virus activity may mainly lie in their similar regulation pathways in cardiovascular systems. These results are also in agreement with our previous findings that pneumonia and influenza mortality risks attributable to influenza did not exhibit a seasonal variation [16]. Extreme heat has been documented to increase blood viscosity through evaporation of body fluid and trigger intravascular coagulation through damaging endothelial cells [24]. Influenza infection has a similar pro-thrombotic effect by inducing inflammation around blood vessels and rupturing atherosclerotic plaques [25]. As a result, mortality risks would be dramatically raised by the stress of both extreme weather and influenza infections. The results suggested that we need extra precautionary measures to reduce influenza infections in people with cardiovascular diseases, especially under the frequent hot and humid weather conditions experienced in the tropical and subtropical areas. Although the experiments of influenza virus transmission between guinea pig hosts found the higher transmission rates occurred under dry air (vapor pressure below 10hPA) [10], our results indicated that the mortality risks associated with influenza under the low vapor pressure environment were lower than the rest of study period. The reason could be the short time of exposure to the very low level of vapor pressure. During our study period there were only 2 and 13 weeks with an average vapor pressure below 10hPA in Hong Kong and Guangzhou, respectively. Most weekly average vapor pressures during the low vapor pressure period were within the range of 10-20hPA, in which the guinea pig experiments showed dramatically reduced transmission rates [10]. In future, we may examine the seasonal variation in influenza effects in other cities to assess whether such a seasonal variation, if common in subtropical and tropical cities, is consistently determined by environmental factors, or by other factors such as host immunity and virus virulence.

The results for the different age groups suggested that the modification effects of environmental factors may mainly lie in the elderly aged over 65 years, as the consistent increasing trend over the low to high vapor pressure periods was only observed in this age group. However, since over 65% of deaths occurred in this age group for both cities (Table 1), the small numbers of weekly death counts in the younger age group (< 65 years) may not have had enough power to allow assessment of effect modification based on the data over the 3 years. A future study with a long study period or a large population may help answer whether young people maybe also expose to higher mortality risks during the hot and humid days.

In this study, we used the quartiles of weekly data in each city, to separately define the periods with normal (middle) and extreme (low and high) weather. Given the difference in weather conditions between these cities, we think that it is not appropriate to use the same cutoff points for temperature or humidity to compare their modification effects on influenza associated mortality, as people living in hot subtropical and tropical regions may adapt well to the year-round hot and humid climate and have a higher threshold for adverse effects of weather. For example, although it is widely accepted that the temperature effects on mortality exhibited a U- or V-shape curve in both temperate and tropical/subtropical areas, the turning point of this curve varied across different cities. A study conducted in 11 cities of the US found that the turning point of temperature for its effects on mortality could range from 18.4°C to 32.4°C [26]. To our best knowledge, so far there are no studies that have ever assessed the effect modification of temperature on influenza effects. Therefore, the commonly adopted cutoff points of city-specific quartiles seem appropriate at this stage [21,27].

There are several limitations in our study. Firstly, our study is based on 3 years of surveillance data which may not have enough power to allow assessment of exposure-response curves for the effects of environmental factors. Nevertheless, our findings did suggest an increasing trend of influenza associated mortality risks across the periods of low, middle and high vapor pressure, although such findings may be applicable only to the warm climates. Secondly, we only investigated the effect modification of environmental factors through a simple interaction model, but there were other unadjusted factors, including host susceptibility and virulence of influenza strains. These factors are unlikely to work independently with environmental factors. Other environmental factors such as ultraviolet radiation [28], rainfall [29] have been proposed to play a role in the regulation of influenza seasonality, although evidence is rather limited compared with the three factors we chose to investigate [30]. Lastly, we did not adjust for the vaccination rate in our model. In 2003, vaccination rate was 191 doses per 1,000 total population in Hong Kong [31], slightly higher than the rate of 129 doses/1,000 total population in Guangzhou [21]. However, it is not clear when people received vaccination; therefore we were unable to assess the role of vaccination in our study.

## Conclusions

This study provides a piece of key evidence to the effect of environmental factors on severity of seasonal influenza under warm climates and helps reveal the mechanism behind global influenza seasonality. It also highlights the need for people with chronic cardiovascular and respiratory conditions to take extra caution against influenza during the hot and humid days in the subtropics.

## Competing interests

JSMP served as ad hoc consultant to pharmaceutical firms Crucell MV and Sanofi Pasteur. Other authors declare that they have no competing interests.

## Authors' contributions

CMW and JSMP initiated the study; PYC, JFH, CQO and APD collected and cleaned the data; LY and KPC conducted the data analysis; LY and CMW drafted the manuscript. All authors read and approved the final manuscript.

## Pre-publication history

The pre-publication history for this paper can be accessed here:

http://www.biomedcentral.com/1471-2334/11/342/prepub

## Supplementary Material

Additional file 1 **Tables S1 and S2; Figures S1 and S2**. Click here for file
