# Supplementary material for: Effect modification of environmental factors on influenza-associated mortality: a time-series study in two Chinese cities
Source: BMC Infect Dis. 2011 Dec 14;11:342. doi: 10.1186/1471-2334-11-342 (PMC3265445; doi:10.1186/1471-2334-11-342)
Supplement: Additional file 1 — Tables S1 and S2; Figures S1 and S2. [file 1471-2334-11-342-S1.DOC]

Table S1. Percentage change (%) of mortality counts associated with 1% increase in influenza, the Hong Kong data of 1998-2006 were used.

| **Disease** | **Percentage change (95% CI)** | | | **Deviance** | **Deviance** | ***p*-value*** |
| --- | --- | --- | --- | --- | --- | --- |
|  | **Low** | **Middle** | **High** | **(main)** | **(interaction)** |  |
|  | **Temperature** | | |  |  |  |
| All-cause | 0.14 | 0.09 | 0.17 | 1098.9 | 1093.3 | 0.059 |
|  | (0.06, 0.22) | (0.00, 0.17) | (0.05, 0.28) |  |  |  |
| CRD | 0.20 | 0.12 | 0.35 | 1129.7 | 1113.3 | <0.001 |
|  | (0.09, 0.32) | (0.00, 0.25) | (0.18, 0.53) |  |  |  |
| P&I | 0.32 | 0.16 | 0.35 | 840.7 | 836.1 | 0.097 |
|  | (0.11, 0.54) | (-0.07, 0.39) | (0.03, 0.67) |  |  |  |
|  | **Relative Humidity** | | |  |  |  |
| All-cause | 0.03 | 0.14 | 0.15 | 1098.9 | 1089.9 | 0.011 |
|  | (-0.08, 0.15) | (0.07, 0.21) | (0.06, 0.24) |  |  |  |
| CRD | 0.11 | 0.22 | 0.23 | 1129.7 | 1125.1 | 0.098 |
|  | (-0.06, 0.28) | (0.11, 0.32) | (0.09, 0.36) |  |  |  |
| P&I | 0.28 | 0.30 | 0.22 | 840.7 | 839.8 | 0.620 |
|  | (-0.04, 0.60) | (0.11, 0.50) | (-0.01, 0.46) |  |  |  |
|  | **Vapor Pressure** | | |  |  |  |
| All-cause | 0.12 | 0.11 | 0.20 | 1098.9 | 1092.4 | 0.038 |
|  | (0.03, 0.20) | (0.03, 0.19) | (0.09, 0.31) |  |  |  |
| CRD | 0.19 | 0.17 | 0.33 | 1129.7 | 1120.6 | 0.011 |
|  | (0.07, 0.31) | (0.05, 0.29) | (0.16, 0.51) |  |  |  |
| P&I | 0.31 | 0.18 | 0.38 | 840.7 | 836.5 | 0.121 |
|  | (0.08, 0.53) | (-0.04, 0.40) | (0.08, 0.68) |  |  |  |

CRD, cardiorespiratory; P&I, pneumonia and influenza; CI, confidence interval.

* p-value was derived from the likelihood ratio test between the interaction and main effect model.

Table S2. Percentage change (%) of mortality counts associated with 1% increase in influenza. The anomalies of metrological factors were used to define different periods.

| **Disease** | **City** | **Percentage change (95% CI)** | | | **Deviance**  **(main)** | **Deviance**  **(interaction)** | ***p*-value*** |
| --- | --- | --- | --- | --- | --- | --- | --- |
|  |  | **Low** | **Middle** | **High** |  |  |  |
|  | | **Temperature** | | |  |  |  |
| All-cause | Guangzhou | -0.11 | 0.22 | 0.44 | 608.3 | 576.6 | <0.001 |
|  |  | (-0.49, 0.27) | (0.00, 0.45) | (0.18, 0.69) |  |  |  |
|  | Hong Kong | 0.10 | 0.17 | 0.34 | 343.9 | 325.3 | <0.001 |
|  |  | (-0.05, 0.24) | (0.04, 0.30) | (0.17, 0.52) |  |  |  |
| CRD | Guangzhou | -0.11 | 0.37 | 0.60 | 642.2 | 613.2 | <0.001 |
|  |  | (-0.65, 0.43) | (0.05, 0.69) | (0.24, 0.96) |  |  |  |
|  | Hong Kong | 0.11 | 0.37 | 0.57 | 320.8 | 293.1 | <0.001 |
|  |  | (-0.09, 0.31) | (0.19, 0.55) | (0.32, 0.81) |  |  |  |
| P&I | Guangzhou | 1.32 | 1.04 | 1.47 | 189.3 | 188.1 | 0.548 |
|  |  | (-0.01, 2.66) | (0.24, 1.84) | (0.61, 2.34) |  |  |  |
|  | Hong Kong | 0.34 | 0.80 | 0.82 | 241.2 | 228.9 | 0.002 |
|  |  | (-0.03, 0.70) | (0.48, 1.12) | (0.39, 1.26) |  |  |  |
|  | | **Relative humidity** | | |  |  |  |
| All-cause | Guangzhou | 0.32 | 0.26 | 0.29 | 608.3 | 607.8 | 0.764 |
|  |  | (-0.08, 0.71) | (0.04, 0.48) | (-0.05, 0.64) |  |  |  |
|  | Hong Kong | 0.13 | 0.17 | 0.18 | 343.9 | 343.4 | 0.789 |
|  |  | (-0.04, 0.31) | (0.04, 0.30) | (-0.09, 0.46) |  |  |  |
| CRD | Guangzhou | 0.57 | 0.38 | 0.41 | 642.2 | 640.1 | 0.346 |
|  |  | (0.02, 1.12) | (0.06, 0.69) | (-0.07, 0.90) |  |  |  |
|  | Hong Kong | 0.30 | 0.28 | 0.17 | 320.8 | 319.8 | 0.597 |
|  |  | (0.06, 0.54) | (0.11, 0.46) | (-0.22, 0.56) |  |  |  |
| P&I | Guangzhou | 1.14 | 1.30 | 0.78 | 189.3 | 188.2 | 0.574 |
|  |  | (-0.23, 2.53) | (0.59, 2.02) | (-0.46, 2.03) |  |  |  |
|  | Hong Kong | 0.44 | 0.67 | 0.53 | 241.2 | 238.6 | 0.276 |
|  |  | (0.00, 0.89) | (0.36, 0.97) | (-0.16, 1.22) |  |  |  |
|  | | **Vapor pressure** | | |  |  |  |
| All-cause | Guangzhou | -0.04 | 0.28 | 0.42 | 608.3 | 582.2 | <0.001 |
|  |  | (-0.37, 0.29) | (0.03, 0.54) | (0.17, 0.66) |  |  |  |
|  | Hong Kong | 0.12 | 0.18 | 0.31 | 343.9 | 333.1 | 0.005 |
|  |  | (-0.02, 0.26) | (0.05, 0.32) | (0.13, 0.50) |  |  |  |
| CRD | Guangzhou | 0.03 | 0.50 | 0.53 | 642.2 | 622.7 | <0.001 |
|  |  | (-0.43, 0.50) | (0.14, 0.87) | (0.18, 0.88) |  |  |  |
|  | Hong Kong | 0.21 | 0.35 | 0.52 | 320.8 | 308.8 | 0.002 |
|  |  | (0.01, 0.40) | (0.16, 0.54) | (0.25, 0.79) |  |  |  |
| P&I | Guangzhou | 1.08 | 1.39 | 1.16 | 189.3 | 188.9 | 0.806 |
|  |  | (-0.06, 2.24) | (0.50, 2.29) | (0.32, 2.01) |  |  |  |
|  | Hong Kong | 0.47 | 0.75 | 0.90 | 241.2 | 234.5 | 0.034 |
|  |  | (0.12, 0.82) | (0.42, 1.08) | (0.44, 1.36) |  |  |  |

CRD, cardiorespiratory; P&I, pneumonia and influenza; CI, confidence interval.

* p-value was derived from the likelihood ratio test between the interaction and main effect model.

Figure S1. Time series plots and partial autocorrelation function plots (PACF) of the residuals from core models for all-cause mortality in Guangzhou (GZ) and Hong Kong (HK).

Figure S2. Sensitivity analysis for different cutoff points of 20^th^, 25^th^ or 30^th^ percentile. The percentage change (%) of mortality counts associated with per 1% increase of influenza virus activity were plotted for the low-, middle- and high- periods of (A) temperature, (B) relative humidity and (C) vapor pressure.
